# Influence of pH and Chloride Concentration on the Corrosion Behavior of Unalloyed Copper in NaCl Solution: A Comparative Study Between the Micro and Macro Scales

**DOI:** 10.3390/ma5122439

**Published:** 2012-11-23

**Authors:** Farzin Arjmand, Annemie Adriaens

**Affiliations:** Department of Analytical Chemistry, Ghent University, Krijgslaan 281-S12, Ghent 9000, Belgium; E-Mail: farzin.arjmandgholenji@ugent.be

**Keywords:** copper, polarization, EIS, pitting corrosion, experimental design

## Abstract

The effects of pH and chloride concentration on the electrochemical corrosion of copper in aqueous sodium chloride (NaCl) media were studied at the micro scale using a microcapillary droplet cell and at the macro scale using a conventional large scale cell. Using an experimental design strategy, electrochemical response surface models of copper *versus* pH and NaCl concentration were constructed with the minimum number of experiments required. Results show that the electrochemical behavior of copper under corrosive media shows significant differences between the micro and macro scale experiments. At the micro scale, the pit initiation of copper occurs at more negative potentials for high NaCl concentrations and alkaline pH values. Also, the micro scale potentiostatic measurements indicate higher stabilised passive currents at high NaCl concentrations and low (acidic) pH values. At the macro scale, the pH is shown to have a greater influence on the corrosion potential. The chloride concentration is the most significant factor in the passive current case while at the micro scale the effect of these two factors on the passive current was found to be the same. The surface morphology of the formed patina on the corroded copper in both micro and macro systems reveal a more significant role of the chloride concentration on the structure and the grain size of the patinas. Finally, micro and macro electrochemical impedance spectroscopy of copper at various NaCl concentrations and pH values demonstrates a different behavior of copper after several potentiodynamic polarization cycles.

## 1. Introduction

It is known that the corrosion mechanism of copper is strongly dependent on the presence of chloride ions. Several attempts to describe the corrosion mechanism of copper in different chloride-containing media have been reported in the literature [1,2,3,4,5,6,7,8,9,10]. The main differences among the available models are the descriptions of the initial electro-dissolution reactions of the bare copper. Three reversible mechanisms have been considered:

(1) $$Cu+2Cl^{-}↔CuCl_{2}^{-}+e^{-}$$

(2) $$\begin{array}{l} Cu↔Cu^{+}+e^{-}\\ Cu^{+}+2Cl^{-}↔CuCl_{2}^{-} \end{array}$$

(3) $$\begin{array}{l} Cu+Cl^{-}↔CuCl+e^{-}\\ CuCl+Cl^{-}↔CuCl_{2}^{-} \end{array}$$

Reactions (1) and (2) show the direct formation of cupric chloride from copper [2,3,5,7,8,9,11], while the third reaction represents the dissolution of copper to cuprous chloride in the first step [1,4,6,7,10].

Despite recent work, the electrochemical behavior of copper at the micro scale still requires further clarification. Information measured using local electrochemical impedance spectroscopy (LEIS) remains scarce [11,12,13,14,15,16,17,18,19,20,21,22,23,24]. Probe electrochemical techniques, using microcapillary cells or micro-electrodes, show key differences compared with macro scale systems, affirming the suitability of these techniques to electroanalytical applications and kinetic studies [24,25,26,27,28,29,30].

It is known that due to the low limiting current, only relatively large pits can be detected by large scale techniques. Moreover, although microelectrodes decrease the measured current density, the limiting current density and the current resolution increase, which improves the electrochemical response in micro systems [31,32]. 

Our previous work [33] showed that miniaturising the measurement area (especially in the case of EIS) results in more detailed information about the corrosion process. Conventional EIS reflects only average information about the electrochemical mechanisms that occur at the electrode/electrolyte interface.

This study investigates the electrochemical corrosion of copper in macro and micro systems in various NaCl solutions. Ranges of pH and chloride concentration promoting local and global corrosion of unalloyed copper are examined, as are conditions promoting breakdown of the passive oxide film.

Linear sweep voltammetry and chronoamperometry are used to determine electrochemical surface plots for copper in NaCl, at both the micro and macro scales. An experimental design strategy known as central composite design (CCD) was implemented to minimize the number of experiments required, and additionally determine which variable (pH or NaCl concentration) has the greater influence on the corrosion of copper. Furthermore, this design strategy was able to reveal correlations between the pH and NaCl concentration.

## 2. Results and Discussion

### 2.1. Potentiodynamic and Potentiostatic Measurements

**Figure 1:**
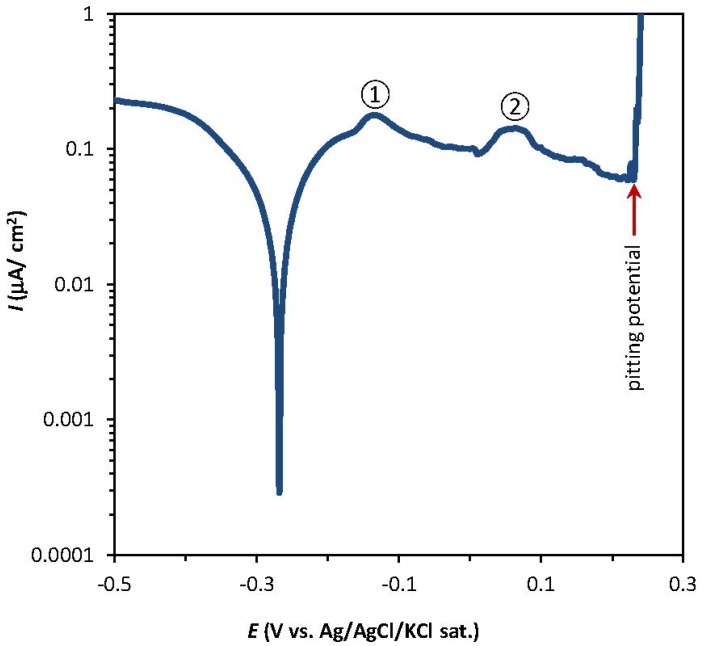
Potentiodynamic polarization curve of pure copper in 3.82 M NaCl (pH = 9.8) obtained using a microcapillary with a tip diameter of ~100 µm. The numbers 1 and 2 indicate the position of the peaks representing the formation of Cu(I) and Cu(II), respectively.

Figure 1 shows a typical potentiodynamic polarization curve of pure copper in 3.82 M NaCl (pH = 9.8) taken at the micro scale level, where the two peaks represent the formation of Cu(I) and Cu(II). The figure also shows the pitting potential, which is the potential where the pitting starts, and is indicated by a rapid rise in the oxidation current. 

**Figure 2:**
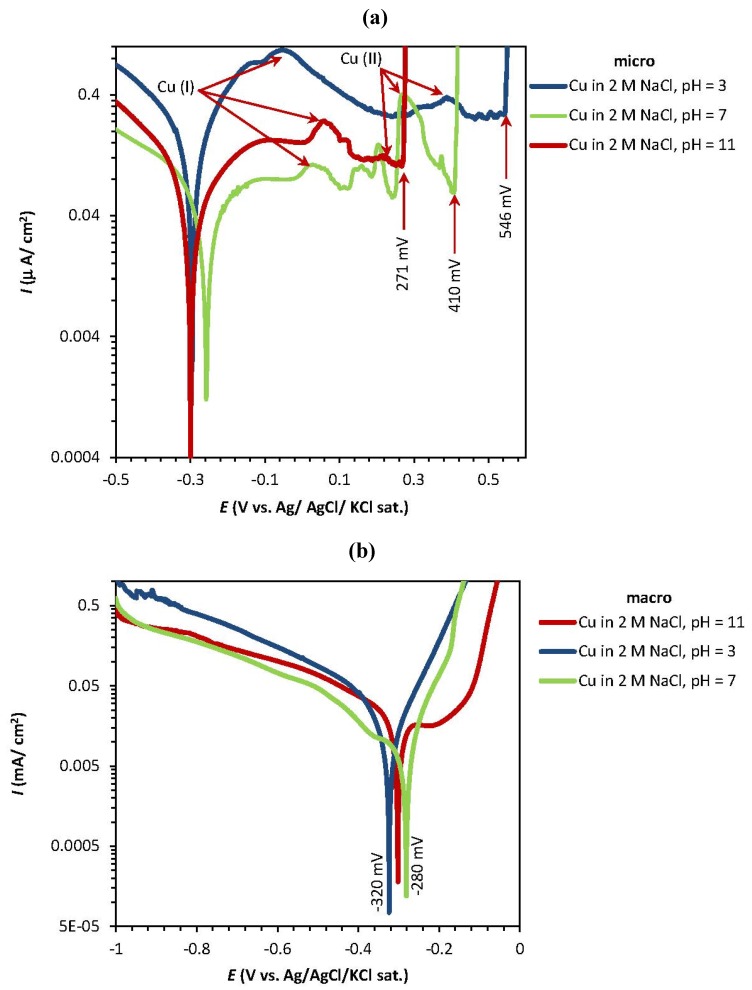

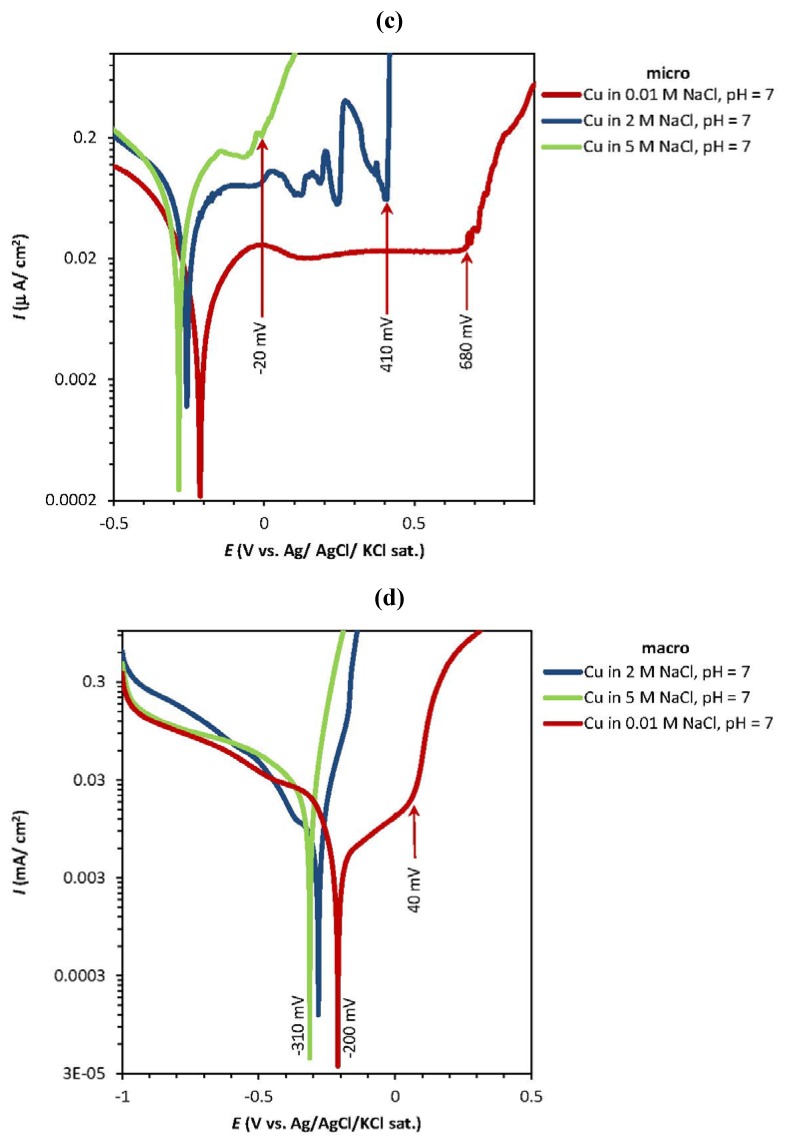
Potentiodynamic polarization curves of pure copper for various (**a**,**b**) pH values and (**c**,**d**) NaCl concentrations at the micro and macro scale. The tip diameter of the used microcapillary is ~100 μm.

Figure 2a shows similar curves taken at different pH values (acidic, neutral, and alkaline). In all cases, peaks 1 and 2 can be clearly identified, which is not the case when using a conventional macro scale setup. In the latter case, two separate anodic peaks can only be distinguished at highly alkaline pH values [34]. 

Figure 2a–d also shows the variations of the pitting and corrosion potential of copper parallel to the variations of the pH and the NaCl concentration at the micro and macro scale. At the micro scale, the pitting potential of copper decreases from 546 to 271 mV/Ag/AgCl with increasing pH values (3, 7, and 11, respectively) (Figure 2a). Higher concentrations of NaCl in neutral pH (7) result in increasingly lower pitting potentials for copper (Figure 2c).

The macro scale Tafel plots of copper in Figure 2b,d reveal a very short passive area in the anodic region, especially for high chloride concentrations. The passive area before the pit nucleation (and therefore the pitting potential) is detectable only for low chloride concentrations (0.01 M). The *E*_corr_ value shifts towards more negative potentials as the chloride concentration is increased.

**Figure 3:**
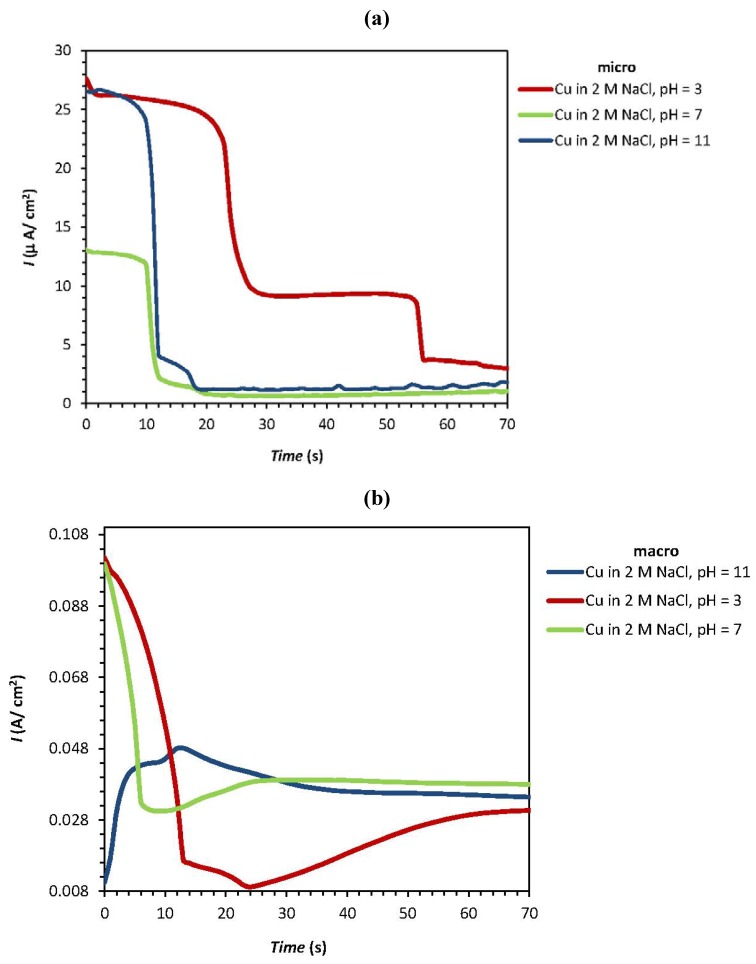

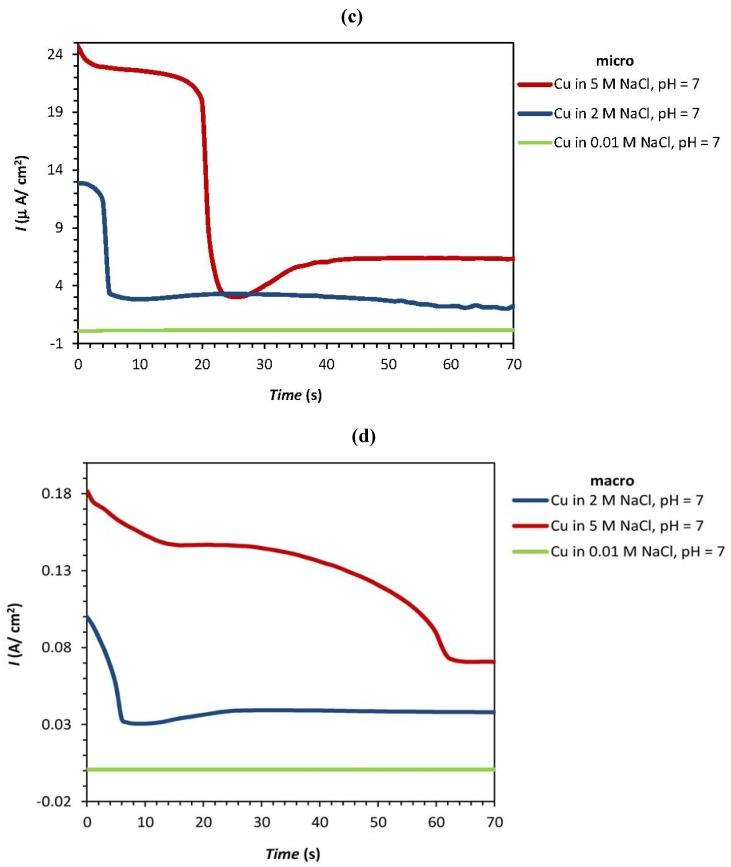
Potentiostatic polarization curves of pure copper for various (**a**,**b**) pH values and (**c**,**d**) NaCl concentrations at the micro and macro scale. The tip diameter of the used microcapillary is ~100 μm.

Figure 3a–d shows the potentiostatic polarization curves of copper in NaCl for different pH values and different chloride concentrations in micro (a and c) and macro (b and d) systems. At the micro scale, the *I*_pass_ of copper shows a sharp decrease at neutral pH (7) to less than 15 µA/cm^2^ whilst the values at acidic and alkaline pH were observed between 25 to 30 µA/cm^2^ (Figure 3a). In addition, higher NaCl concentrations show higher passive currents for the micro system (Figure 3c). Figure 3b,d show that higher *I*_pass_ values are obtained at neutral pH (7) and higher chloride concentrations, and that changes in pH have no significant effect on the response, especially in acidic and neutral pHs.

The corrosion of copper with an oxide film typically starts with the removal of the passive oxide layer, followed by oxidation of the copper to different oxidation states. This process forms corroded pits, which are detected through the rapid increase of the oxidation current. Figure 2a,c and Figure 3a,c therefore suggest that in micro scale systems the pit corrosion of pure copper in sodium chloride solutions occurs more readily at higher NaCl concentrations and alkaline pH values; however, the passive current of oxide film breakdown is higher at acidic pH and high NaCl concentrations. In macro scale systems the corrosion parameters of copper are dominated by the chloride concentration.

Two main questions remain. Which factor is more significant in each system? What is the correlation between these two factors? To answer these questions, an experiment was designed using the central composite design method in order to create a clear surface map of the *E*_corr(macro)_, *E*_pit(micro)_ and *I*_pass(micro/macro)_ variations *versus* pH and sodium chloride concentration. The significance of each variable was investigated. This central composite design consists of 12 measurements for each response (*E*_pit(micro)_, *E*_corr(macro)_ and *I*_pass(micro/macro)_). Table 1 shows the levels of the coded and actual experimental variables that were tested, and the corresponding response of each experiment. 

**Table 1:** Design matrix and relative *E*_pit(micro)_, *I*_pass(micro/macro)_ and *E*_corr(macro)_ values in the central composite design for two factors: NaCl concentration (*molar*) and pH. The letter “a” in the experiment column indicates the replicate measurements.

Experiment	*F*_1_: [NaCl] (M)	*F*_2_: pH	*E*_pit (micro)_ (V)	*E*_corr(macro)_ (V)	*I*_pass(micro)_ (µA/cm^2^)	*I*_pass(macro)_ (A/cm^2^)
*1*	0	2	0.186	−0.302	26.5	0.0105
*2*	0	−2	0.167	−0.324	27.15	0.102
*3^a^*	0	0	0.410	−0.281	13.1	0.099
*4*	1.414	−1.414	0.374	−0.310	44.35	0.142
*5*	2	0	−0.063	−0.313	24.7	0.182
*6^a^*	0	0	0.364	−0.287	12.9	0.0864
*7^a^*	0	0	0.373	−0.280	12.9	0.0946
*8*	−1.414	1.414	0.539	−0.247	7.83	0.0314
*9*	−1.414	−1.414	0.311	−0.239	9	0.0327
*10*	−2	0	0.781	−0.210	0.102	0.0007
*11*	1.414	1.414	0.241	−0.332	22.65	0.163
*12^a^*	0	0	0.396	−0.262	13.25	0.080
*Coded value (*−*2)*	*0.01*	*3*	–	–	–	–
*Coded value (*−*1)*	*0.59*	*4.1*	–	–	–	–
*Coded value (0)*	*2*	*7*	–	–	–	–
*Coded value (+1)*	*3.828*	*9.8*	–	–	–	–
*Coded value (+2)*	*5*	*11*	–	–	–	–

**Figure 4:**
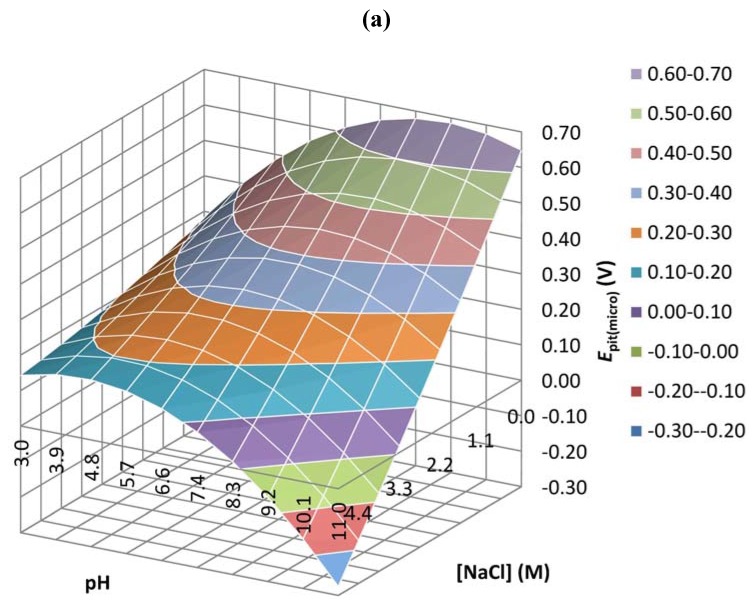

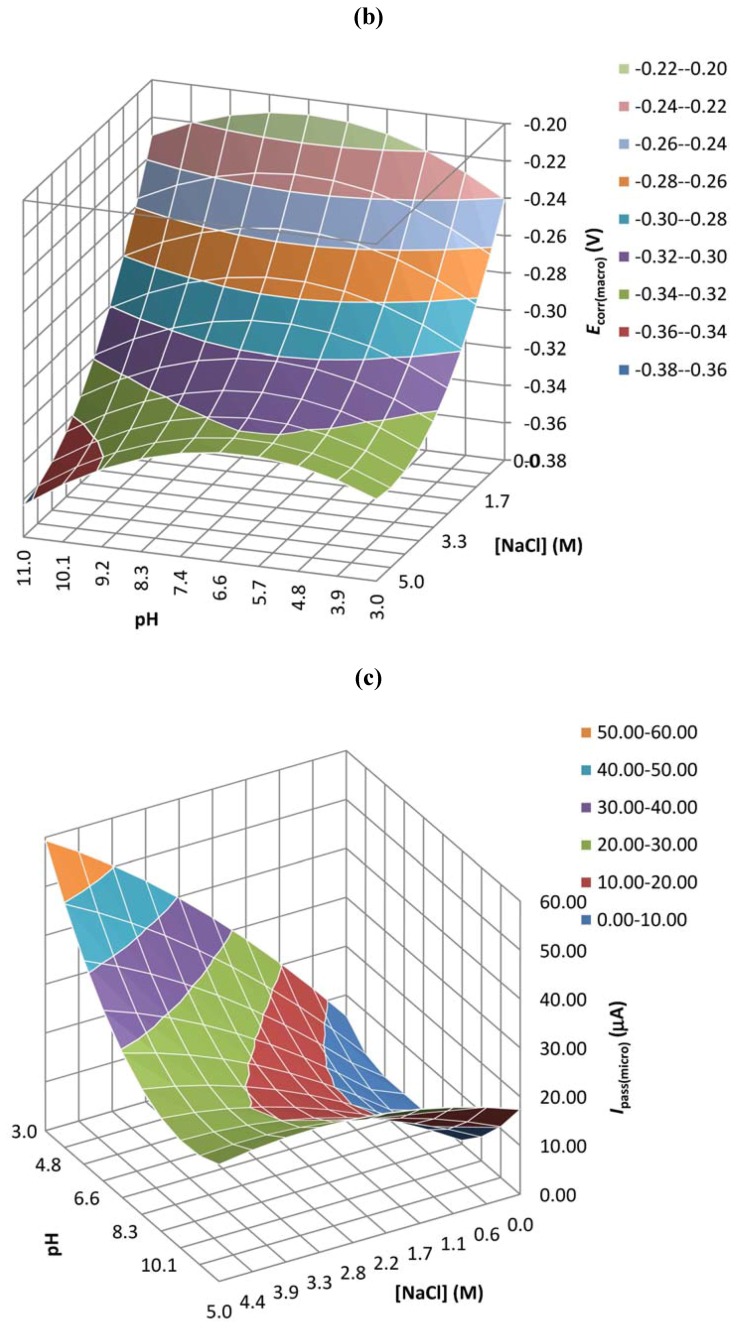

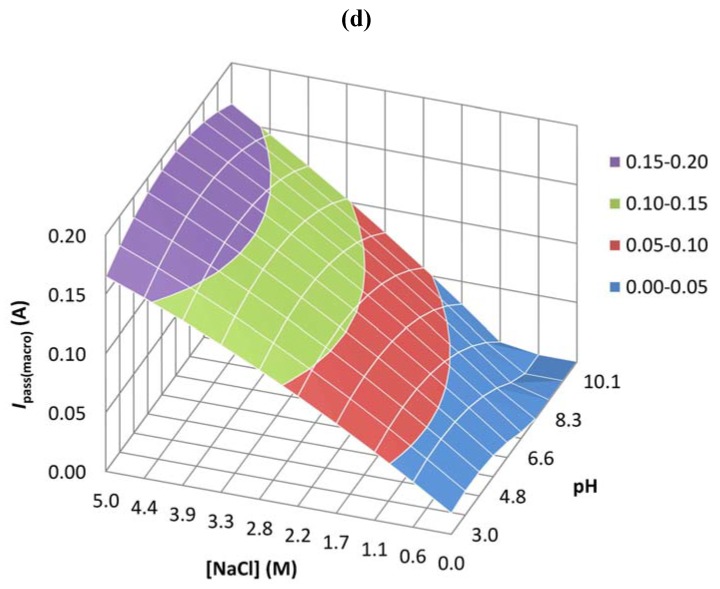
Response surface plots obtained from the central composite design by plotting pH *vs.* NaCl concentration. (**a**) *E*_pit(micro)_; (**b**) *E*_corr(macro)_; (**c**) *I*_pass(micro)_ and (**d**) *I*_pass(macro)_.

Figure 4a,c shows respectively the response surface plots of the pitting potential and the stabilised passive current of copper *versus* pH and the NaCl concentration on the micro scale level. 

The pitting potential surface plot (Figure 4a) demonstrates that the most corrosive medium for copper is a high NaCl concentration (~5 M) and a highly alkaline pH (~11) where *E*_pit_ = −0.3 to −0.2 V/Ag/AgCl. The highest pitting potential, on the other hand, is observed at neutral pH values and low chloride concentrations (*E*_pit_ = 0.6 to 0.7 V/Ag/AgCl). Figure 4c shows the highest passive current for acidic pH values (pH = 3) and high chloride concentrations (~5 M). The passive current increases from 5 to 20 µA at low NaCl concentration (<0.5 M) as the pH increases (up to 11).

Figure 4b,d shows the variation of *E*_corr_ and *I*_pass_, respectively, against pH and NaCl concentration at the macro scale. The lowest corrosion potential is observed at high chloride concentrations (~5 M) and highly alkaline pH (~11). This is similar to the *E*_pit_ variation observed at the micro scale. The highest *I*_pass_, however, was observed at high chloride concentrations over a wide range of pH values.

The *p*-values obtained from the ANOVA are shown in Table 2. In statistical analyses, *p*-values are the most commonly used tool to measure evidence against a hypothesis model. The *p*-value is a probability, with a value ranging from 0 to 1 indicating the possibility of observing a difference between the real value and the estimated value [35]. Factors with lower *p*-value are therefore more significant.

**Table 2:** *p*-values for each response.

Coefficient	*E*_pit(micro)_	*I*_pass(micro)_	*E*_corr(macro)_	*I*_pass(macro)_
*b*_0_	0.647	0.029	0.000	0.905
*b*_1_	0.910	0.003	0.021	0.238
*b*_2_	0.119	0.005	0.017	0.479
*b*_3_	0.894	0.200	0.024	0.632
*b*_4_	0.189	0.002	0.018	0.240
*b*_5_	0.260	0.002	0.496	0.540

Table 2 shows that in the case of the micro scale pit initiation of copper, the obtained *p*-values for factors *F*_1_ and *F*_2_ are 0.910 and 0.119, respectively. This confirms a greater influence of pH variation on the pitting potential of copper. The second lowest *p*-value (0.189) was observed for the fifth term (*b*_4_ × pH × pH). At the micro scale, the *p*-values for the passive current are 0.003 and 0.005 for the first and second factors, respectively. This suggests that the influence of the NaCl concentration and pH on the response is approximately equivalent. In this case the lowest *p*-values (0.002) were observed for the interaction between the second factor (*b*_4_ × pH × pH) and the interaction of the first and second factors (*b*_5_ × pH × [NaCl]). The *p*-value for pH itself (0.005) was also significant.

On the macro scale, the *p*-values for *E*_corr_ show a higher significance for pH (*p*-value: 0.017). Here also the second important term is pH × pH (*p*-value: 0.018) and the third important term is NaCl concentration (*p*-value: 0.021). In the case of *I*_pass(macro)_ the NaCl concentration shows a high significance (*p*-value: 0.238). It can be concluded that pH has a greater influence on the *E*_corr_ variations, although the response variation shows a steeper slope on the NaCl axis at the macro scale.

**Figure 5:**
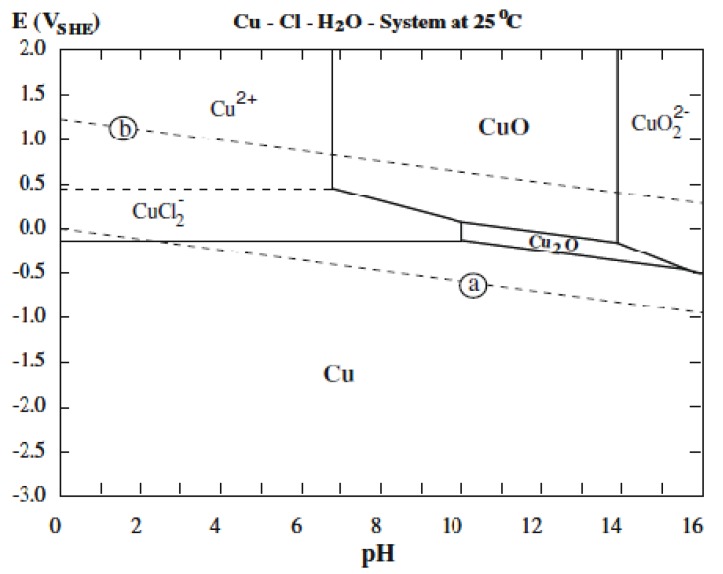
Potential–pH (Pourbaix) diagram of pure copper in the presence of 1 M NaCl solution. The potentials are *vs.* the standard hydrogen electrode [34].

A potential–pH diagram of copper in NaCl solution may be useful to investigate the composition of the corrosion products of copper at different pH values. Figure 5 represents the Pourbaix diagram of copper as reported by Alfantazi in 2009 [34]. It demonstrates that the major copper corrosion products in NaCl media from acidic pH to neutral pH depend on the applied potential and are either CuCl_2_^−^ or Cu^2+^, while for alkaline pH values (~7–14) Cu_2_O and CuO represent the majority of the corrosion products. Finally, at pH values higher than 14 and potentials higher than 0 V/SHE, CuO_2_^2−^ is the only expected compound of copper corrosion.

### 2.2. Surface Morphology

**Figure 6:**
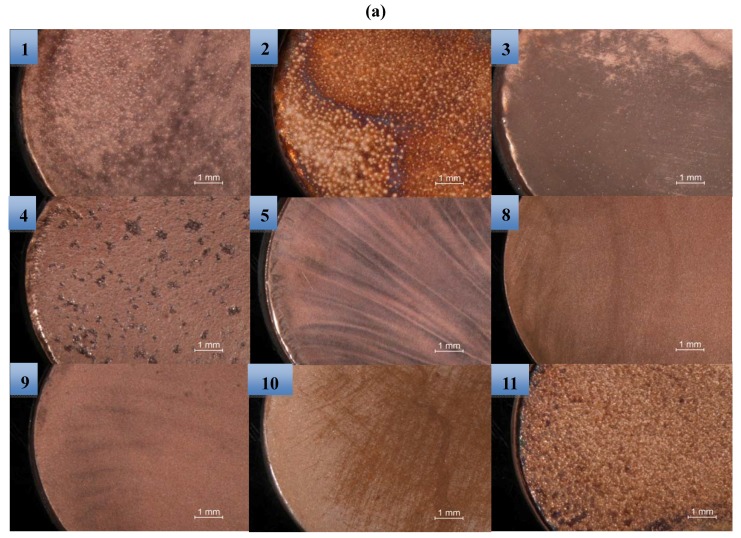

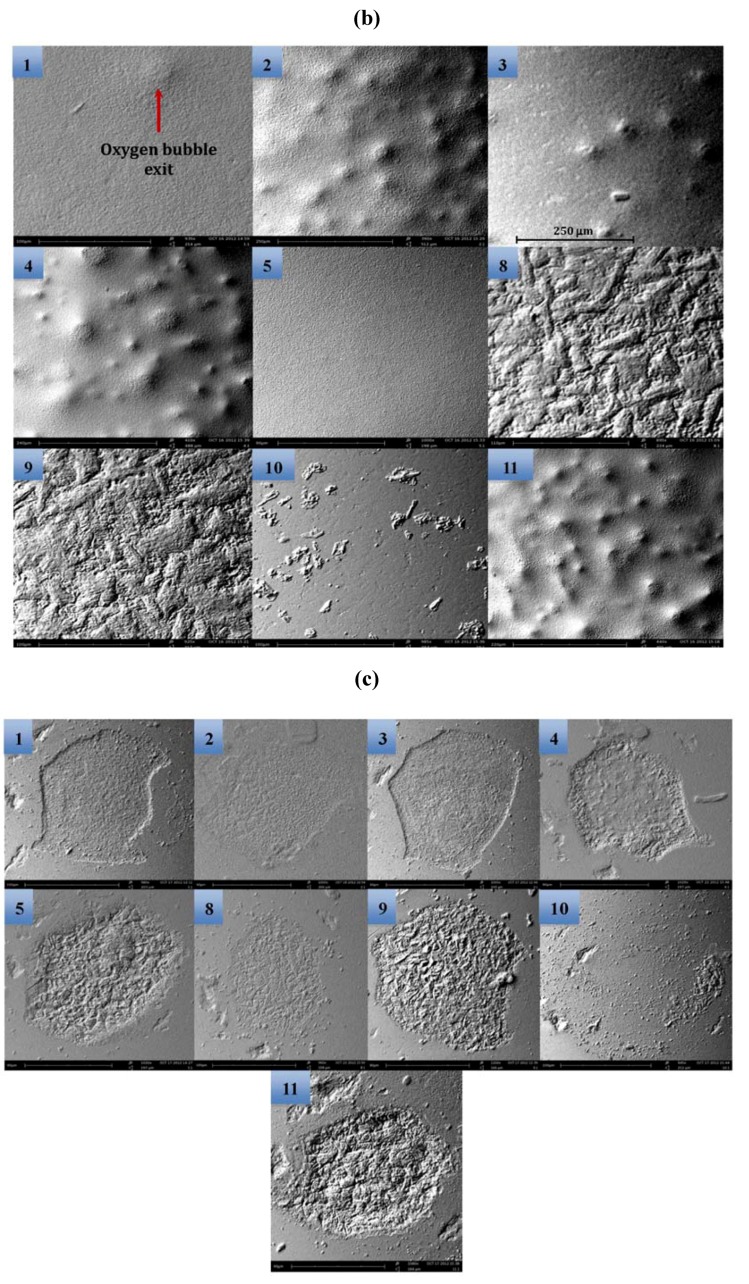
(**a**) Optical images of the anodically polarized pure copper in various NaCl solutions (different concentrations and pH values) on a macro scale; (**b**) related SEM images and (**c**) SEM images of the corroded copper samples using a microcapillary cell. The numbers indicate the designed experiments mentioned in Table 1. Scan rate: macro: 1 and micro 10 mV/s. Tip diameter of the used microcapillary: ~100 μm.

The surface morphology of the various copper samples after potentiodynamic polarization is shown in Figure 6 both for the macro scale (a and b) as for the micro scale (c). Comparison of the corroded layers between images number 8, 9 and 11 in Figure 6 (a and b) reveals that it is mostly chloride concentration which changes the surface morphology of the formed patina. In the almost saturated concentration of 5.0 M, a thick layer of patina covers the surface (Figure 6 (a and b), image 5), while in the 0.01 M solution a thin patina layer is formed in parts of the sample (Figure 6 (a and b), image 10). Moreover, strangely in the 0.59 M solution, in both acidic and alkaline pH values (Figure 6 (a and b), images 8 and 9) the formed patina layers on the copper surface show a different morphology with large grains. In addition, for images 2, 4 and 11 (Figure 6b) inflated points under the patina are observed, which can be attributed to formed oxygen bubbles during the anodic polarization of the surface. In the micro images some differences in the patina morphology are observed. For instance images 5b and 11b show different shapes of patinas compared to the macro images. However, as macro images, here also, the general morphology of the formed patinas under the microcapillary supports the idea that it is chloride concentration which has the more significant role in surface morphology of the corroded copper.

### 2.3. Electrochemical Impedance Spectroscopy (EIS)

**Figure 7:**
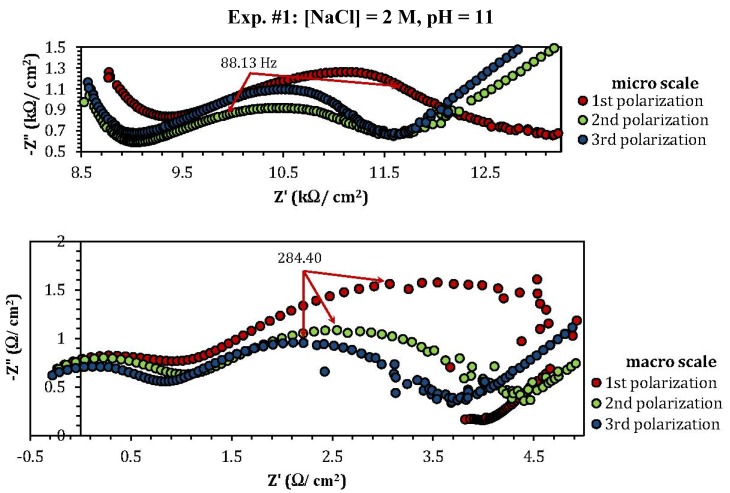

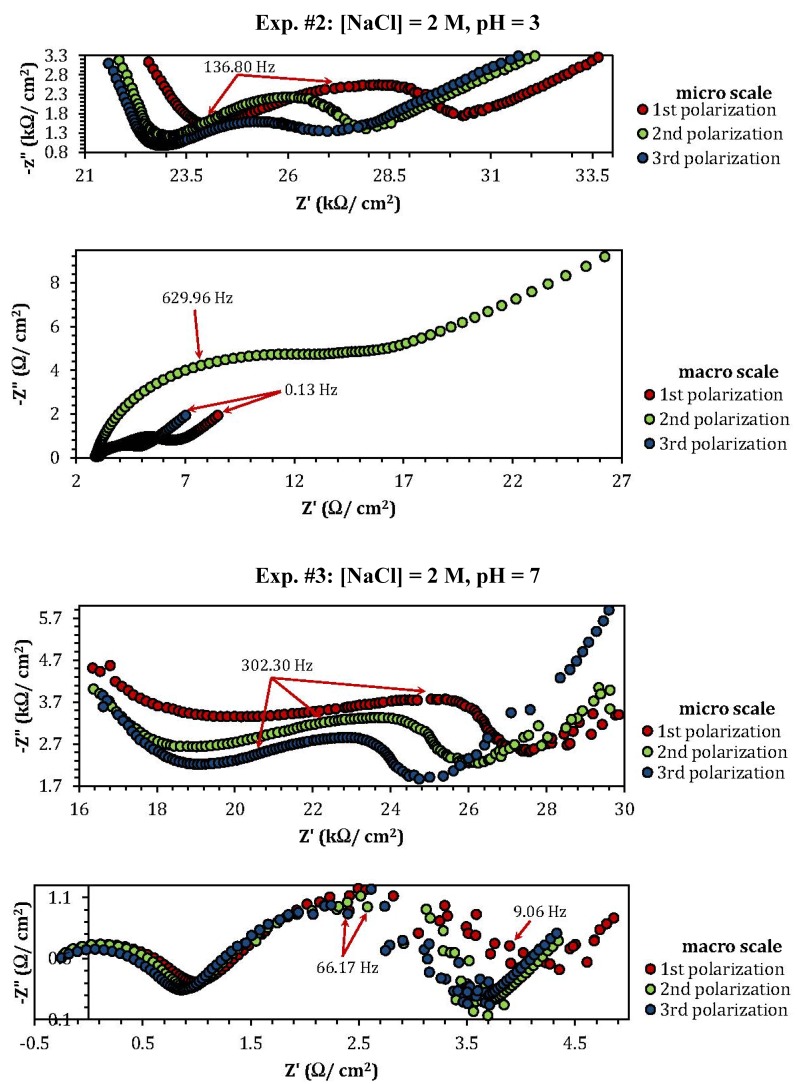

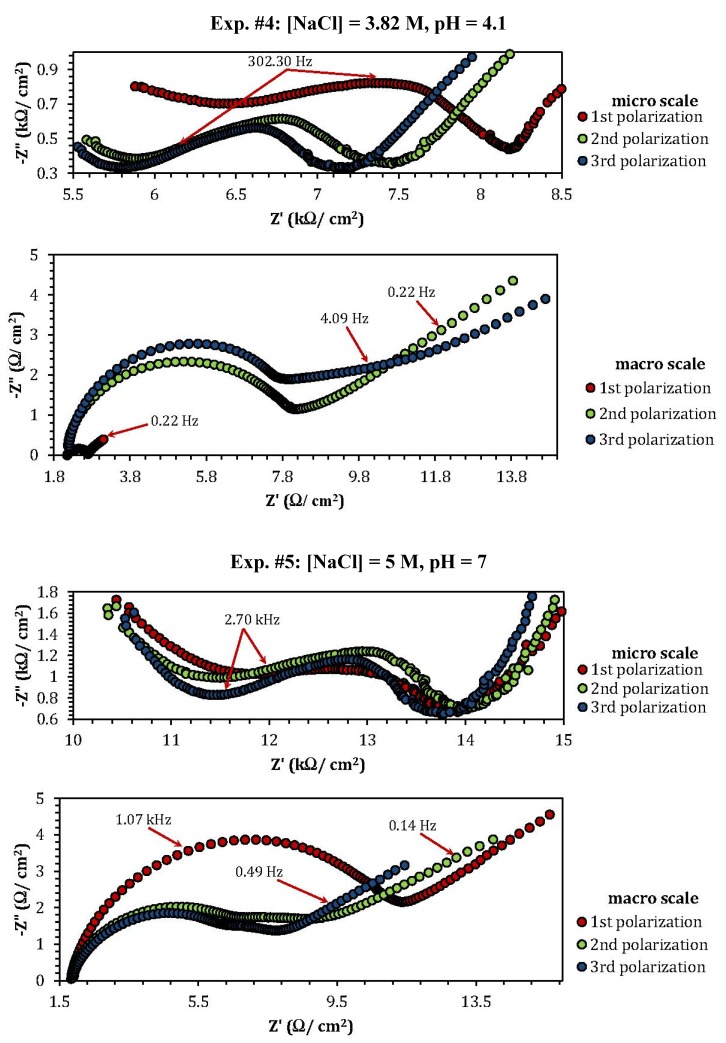

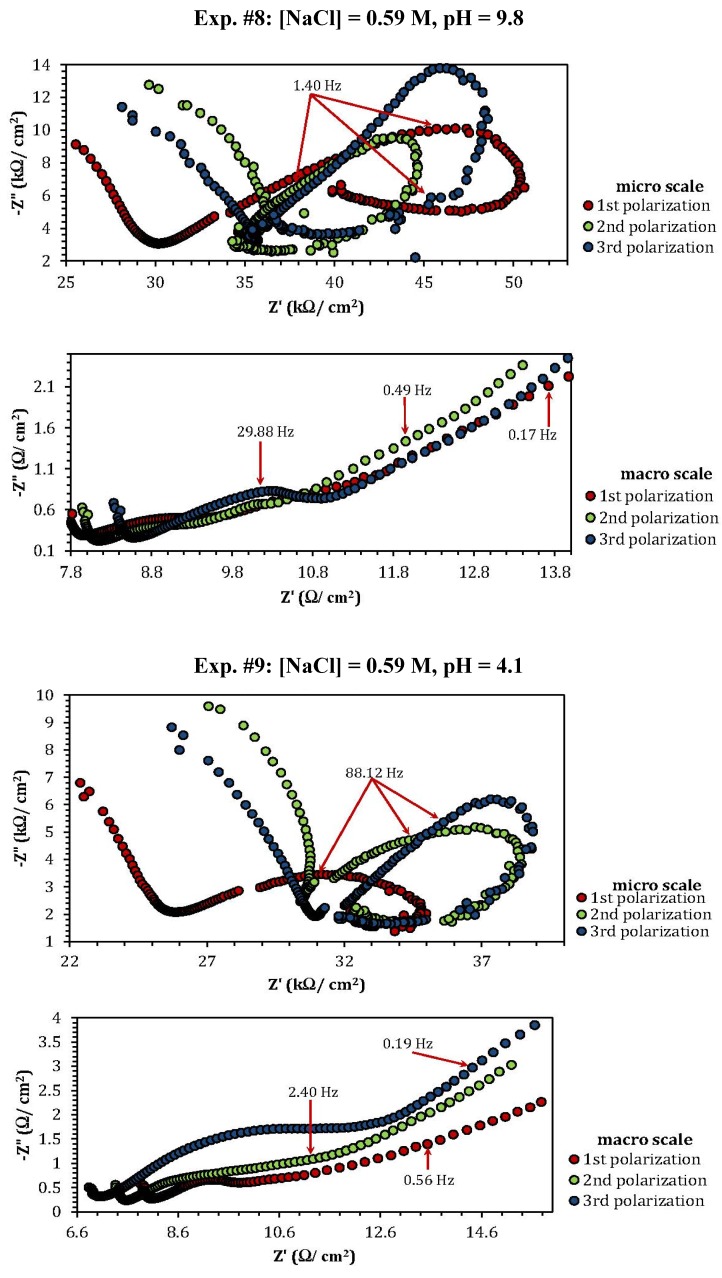

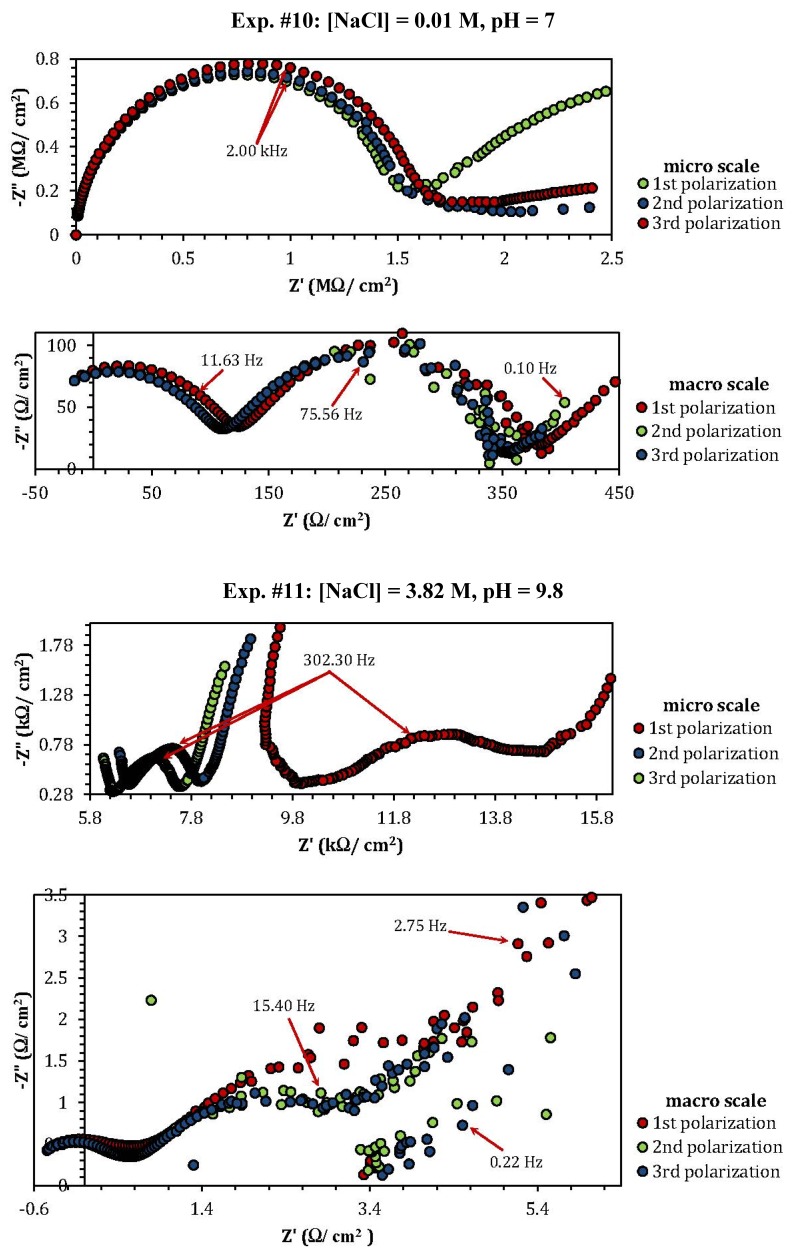
Micro and macro scale Nyquist diagrams of pure copper measured in NaCl solutions at different concentrations and pH values after three continuously potentiodynamic polarizations.

Figure 7 shows the Nyquist graphs, taken both on micro and macro scale, of copper after three continuous polarization runs in a NaCl solution with different pH values and NaCl concentrations. This series of experiments was performed under the same experimental design conditions as those listed in Table 1. A first distinction between the micro and macro for all experiments is the significant difference in the measured impedance range (micro: kΩ/cm^2^ and macro: Ω/cm^2^), which is attributed to a smaller current density in the micro system, resulting in larger impedance values. Moreover, the micro scale graph of experiment 10 ([NaCl] = 0.01 M, pH = 7) shows high impedance values, reaching up to 1 MΩ, in comparison to all other micro scale impedance results, which are in the kΩ range. This can be interpreted as a higher corrosion resistance of copper at lower NaCl concentrations.

**Figure 8:**
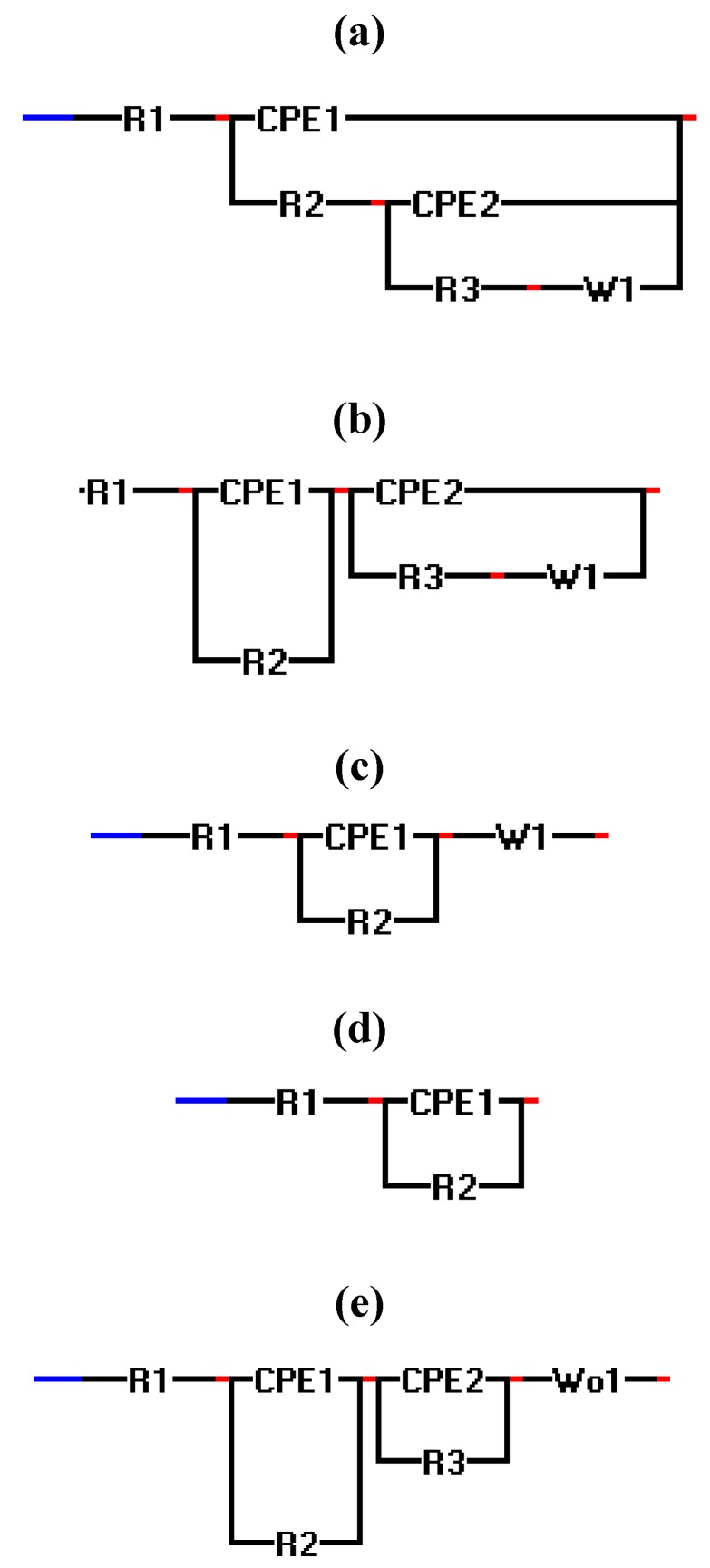
Electrochemical equivalent circuits to fit the EIS data.

The electrochemical equivalent circuits fitted with the micro and macro scale Nyquist graphs of Figure 7 are shown in Figure 8. In this figure R_1_ is the solution resistance, R_2_ the charge-transfer resistance, R_3_ the polarization resistance, CPE the constant phase element and W is the Warburg diffusive impedance. In this figure, model 8c is usually used to describe electrode processes, in which both kinetics and diffusion are significant [36,37,38].

Table 3 lists every Nyquist spectrum of Figure 7 with its relative electrochemical circuit represented in Figure 8. Although it is not easy to construct an electrochemical circuit model for the micro scale Nyquist graphs of experiments 8 ([NaCl] = 0.59 M, pH = 9.8) and 9 ([NaCl] = 0.59 M, pH = 4.1), the similarity between the two experiments, suggests that the NaCl concentration has a greater influence on the formation procedure of the corroded layers on the copper. The Nyquist graphs of these experiments also show a relaxation impedance (at low frequencies), which indicates that the active surface area, where the reactions occur, is small compared to the passive surface area under the microcapillary. Furthermore, Figure 7 shows that after the first potentiodynamic polarization of the surface, depending on the formed patina layer during the second and the third polarizations the measured impedance value can increase or decrease.

**Table 3:** Overview of the EIS experiments with their electrochemical equivalent circuits shown in Figure 8.

Experiment	Polarization 1	Polarization 2	Polarization 3
1–5_(micro scale)_	a	a	a
10_(micro scale)_	c	d	d
11_(micro scale)_	a	a	b
2,5,8,9_(macro scale)_	a	a	a
4_(macro scale)_	c	a	a
1,3,10,11_(macro scale)_	e	e	e

Table 4 lists the maximum values of the imaginary impedance shown in Figure 7 measured at the peak area of each spectrum after the first potentiodynamic polarization. The results show a minimum Z" value for experiment 4 ([NaCl] = 3.8 M, pH = 4.1) and a maximum Z" value for experiment 10 ([NaCl] = 0.01 M, pH = 7) and this for both micro and macro experiments. This is in good agreement with Figure 4c (micro scale) where the maximum *I*_pass_ is observed for low acidic pH values and high chloride concentrations and the minimum *I*_pass_ is observed for neutral pH values and low chloride concentrations, something that cannot be concluded using the data represented in Figure 4d (macro scale).

**Table 4:** Imaginary impedance values (Z") measured at the peak area of each spectrum (Figure 7) after the first potentiodynamic polarization.

Experiment	Z"_micro scale_ (kΩ)	Z"_macro scale_ (Ω)
1	1.26	1.57
2	2.53	0.8
3	3.76	1.17
*4*	*0.8 (min)*	*0.1 (min)*
5	1.06	3.86
8	10	0.48
9	3.5	0.75
*10*	*0.7 (*M*Ω) (max)*	*102 (max)*
11	0.88	1.75

## 3. Experimental Section 

### 3.1. Chemicals

Sodium chloride solutions were prepared using analytical grade NaCl powder (Fluka). The prepared concentrations and pH values ranged from 0.01 to 5 M and 3 to 11, respectively. The pH was measured using a digital pH meter (ORION, model: 420 A) and adjusted with NaOH (4 M) or HCl (37%) as necessary. The total volume of each solution was 50 mL, of which 10 mL was used for the measurements.

### 3.2. Specimen and Surface Preparation

All experiments were performed on pure copper coupons with a diameter of 12.5 mm and a thickness of 2 mm (Goodfellow Cambrige Ltd., 99.9% purity, temper: half hard). The coupons were ground with silicon carbide paper down to 600 grit and then polished with a polishing cloth (MicroCloth, Buehler) using 1.0 µm and 0.5 µm alumina in sequence. Finally, samples were washed with distilled water and rinsed ultrasonically in ethanol for 5 min.

### 3.3. Preparation of the Microcapillaries with a Silicone Gasket

**Figure 9:**
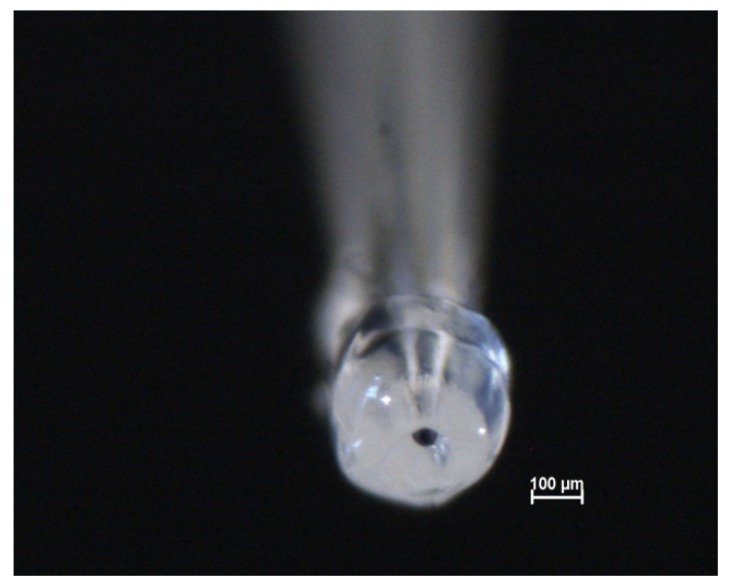
Optical image of a microcapillary with a silicone gasket.

The microcapillaries were produced by heating glass Pasteur pipettes (2 mL) until their glass melting point, after which they were gently drawn, resulting in finer pipettes with different tip diameters. In this study the tip diameter was ~100 µm. The microcapillary tips were ground using first 600 and then 1200 grit silicon carbide paper to ensure a flat surface on which a silicone gasket was attached. The gasket prevents leakage of the electrolyte through crevices between the microcapillary and the specimen surface. The silicone gasket was prepared by dipping the capillary tips into silicone rubber, and flushing a stream of nitrogen through the microcapillary to keep the tip of the capillaries open without destroying the gasket. An optical microscope was used to monitor the procedure. Repeating this process two or three times allows thin layers of silicone to be applied onto the tip of the capillaries (Figure 9).

### 3.4. Electrochemical Set-Up

**Figure 10:**
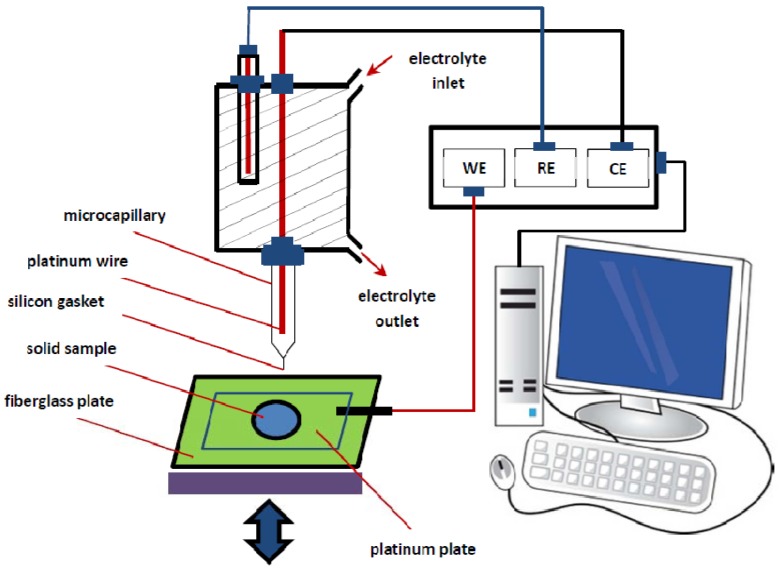
Schematic drawing of the set-up of the capillary-based droplet cell.

Local electrochemical measurements were conducted using a homemade microcapillary cell (Figure 10) attached to an Autolab Eco Chemiepotentiostat (PGSTAT 10). The setup used in this study is based on a common three-electrode system: a thin platinum wire counter electrode, a saturated Ag/AgCl (4.8 mol/L) reference electrode, and a sealed microcapillary with a silicone gasket (tip diameter of ~100 μm). The microcapillary touches only a small part of the copper coupon placed on a platinum plate, which together form the working electrode. The platinum plate itself is attached to a fibreglass plate with a number of holes and plastic screws, which makes it possible to fix samples of different sizes prior to measurement (not shown in Figure 10). Secondary electron images of the surface were taken using a FEI Phenom electron microscope.

The microcapillary is filled with the electrolyte (in this case one of various NaCl solutions). For the macro scale measurements, a conventional electrochemical cell with a saturated Ag/AgCl (4.8 mol/L) reference electrode and a platinum plate as a counter electrode was used.

The Tafel plot measurements were performed using linear sweep voltammetry, in the potential range of −1 to 1 V/Ag/AgCl with a scan rate of 10 and 1 mV/s for the micro scale and macro scale measurements, respectively. The high scan rate for the micro scale measurements was selected to avoid possible cell leakage or blockage of the microcapillary. This strategy has been reported in other studies [16,39]. The potentiostatic measurements were performed at 250 mV/Ag/AgCl with a duration of 200 seconds for both micro and macro systems. Both micro and macro scale EIS measurements were performed in a frequency range of 50 kHz to 0.1 Hz (amplitude of 25 mV/Ag/AgCl at open circuit potential). Nyquist plots of the sample were derived from the potentiodynamic polarization of the surface. The polarization was performed using linear sweep voltammetry in a potential range of −1.5 and 1.3 V/Ag/AgCl and with a scan rate of 25 mV/s. In the case of micro scale measurements a Nyquist spectrum was recorded after each polarization at the same location without removing the microcapillary tip from the surface of the copper sample. This polarization–Nyquist plot procedure was repeated three times.

### 3.5. Experimental Design

A Box-Wilson central composite design [40] was implemented in order to design a short series of experiments to study the pitting potential, corrosion potential, and stabilised passive current of copper in sodium chloride solution. A wide range of concentrations (0.01 to 5 M) and pH values (3 to 11) was investigated at both the micro and macro scales. The central composite design enables an analysis of the correlation between these factors and reveals likely interactions between pH and sodium chloride concentration.

The polynomial equations, response surface, and central design for a particular response were obtained using the statistical software package Essential Regression 97 [41,42]. For an experimental design with two factors, the model includes linear, quadratic, and cross terms that may be expressed as follows:

(4) $$Response=b_{0}+b_{1}\times F_{1}+b_{2}\times F_{2}+b_{3}\times F_{1}\times F_{1}+b_{4}\times F_{2}\times F_{2}+b_{5}\times F_{1}\times F_{2}$$

The response is either the pitting potential (*E*_pit_), the corrosion potential (*E*_corr_), or the passive current (*I*_pass_). *F*_1_ and *F*_2_ are the variable parameters (NaCl concentration and pH, respectively), and *b*_0_ through *b*_5_ are the coefficient values obtained through a multivariate linear regression. The term *b*_0_ indicates the intercept, which is used in calculating the error. The statistical significance of the predicted model was evaluated by an analysis of variance (ANOVA) and least square technique. Replicates (*n* = 4) of the central points were performed to estimate the experimental error.

## 4. Conclusions

A capillary-based microdroplet cell is a powerful tool to investigate corrosion processes and pit initiation at the micro scale, something which is not feasible with conventional macro scale techniques. Although miniaturisation of the measurement area decreases the measured current density down to the range of micro or even nano amperes, it simultaneously enhances the limiting current and therefore improves the resolution of the electrochemical responses compared to macro scale systems. In addition the combination of chemometrics and high-resolution micro data provides additional information that can facilitate the investigation of the corrosion behavior of electroactive solids. Both methods are combined in this work to study the electrochemical corrosion of copper. The influence of two factors (concentration and pH) at the micro and macro scales was compared. Surface plots were obtained based on the recorded electrochemical data after performing an essential regression. Using these plots, one can estimate the *E*_corr(macro)_, *E*_pit(micro)_ and *I*_pass(micro/macro)_ of copper for a wide range of pH and NaCl concentration values. 
